# Mix24X, a Lab-Assembled Reference to Evaluate Interpretation Procedures for Tandem Mass Spectrometry Proteotyping of Complex Samples

**DOI:** 10.3390/ijms24108634

**Published:** 2023-05-11

**Authors:** Charlotte Mappa, Béatrice Alpha-Bazin, Olivier Pible, Jean Armengaud

**Affiliations:** 1 Département Médicaments et Technologies pour la Santé (DMTS), Université Paris-Saclay, CEA, INRAE, SPI, 30200 Bagnols-sur-Cèze, Franceolivier.pible@cea.fr (O.P.); 2 Laboratoire Innovations Technologiques Pour la Détection et le Diagnostic (Li2D), Université de Montpellier, 30207 Bagnols sur Cèze, France

**Keywords:** high-resolution datasets, metaproteomics, microbiota reference, complex sample, proteotyping, tandem mass spectrometry

## Abstract

Correct identification of the microorganisms present in a complex sample is a crucial issue. Proteotyping based on tandem mass spectrometry can help establish an inventory of organisms present in a sample. Evaluation of bioinformatics strategies and tools for mining the recorded datasets is essential to establish confidence in the results obtained and to improve these pipelines in terms of sensitivity and accuracy. Here, we propose several tandem mass spectrometry datasets recorded on an artificial reference consortium comprising 24 bacterial species. This assemblage of environmental and pathogenic bacteria covers 20 different genera and 5 bacterial phyla. The dataset comprises difficult cases, such as the *Shigella flexneri* species, which is closely related to Escherichia coli, and several highly sequenced clades. Different acquisition strategies simulate real-life scenarios: from rapid survey sampling to exhaustive analysis. We provide access to individual proteomes of each bacterium separately to provide a rational basis for evaluating the assignment strategy of MS/MS spectra when recorded from complex mixtures. This resource should provide an interesting common reference for developers who wish to compare their proteotyping tools and for those interested in evaluating protein assignment when dealing with complex samples, such as microbiomes.

## 1. Introduction

Whole-cell matrix-assisted laser desorption ionization-time of flight mass spectrometry (MALDI-TOF MS) has proven to be a powerful methodology to rapidly identify microbial isolates [1]. Unfortunately, its performance is compromised when the sample corresponds to a pathogen in the presence of a matrix or a complex mixture of microorganisms, as is the case for microbiomes. Proteotyping based on tandem mass spectrometry has recently gained momentum for the classification and identification of microorganisms [2,3]. This technology based on the analysis of tryptic peptides obtained from proteins extracted from samples allows strain-level typing of pathogens [4], and the rapid identification of atypical isolates for which no data has been previously recorded, as successfully illustrated with the taxonomical identification of new strains from various environments [5,6]. It also allows the identification of microorganisms from more complex samples, such as biofilms [7] and water [8]. In addition, its routine application for clinical diagnostics can be considered because the methodology is fast to implement [9], high throughput [10], and is sensitive [11]. More recently, this approach has been used to identify specific biothreats from hare carcasses [12], traces of human remains and microorganisms from an ancient relic [13], species out of archaeological bones [14], and even ancient coronaviruses from the dental pulp of individuals buried in the 16th century [15].

Currently, several pipelines have been proposed to interpret the identified peptides and, with this information in hand, trace them back to the organisms that produced the corresponding proteins. The Unipept tool identifies the most likely organisms explaining the peptides based on the lowest common ancestor approach [16]. TCUP directly compares peptide sequences to a comprehensive database of microorganisms [17]. ProteoClade considers taxon-specific peptide sequences found in the queried database [18]. TaxIT specialized for pathogenic single-organism samples is based on iterative searches [19]. Finally, MiCld calculates complex scores to sort out the most relevant taxa [20]. This software also allows the identification of antibiotic resistance proteins [21] and the estimation of the biomass of microorganisms [22]. Identifying the taxa present in a microbiome sample is a key step to focus the metaproteomic search much more narrowly, but the wide diversity of organisms present in such a sample can be a significant challenge [23]. Improved strategies to better identify the taxa present in these samples, while limiting false positives, should be proposed.

The value of models representative of environmental microbial systems for improving experimental protocols and bioinformatics procedures in metaproteomics has been discussed recently [24]. Spiking known bacteria into complex samples, such as fecal material, has proven useful in evaluating database search procedures [25]. Interestingly, a laboratory-assembled microbial mixture comprising nine microorganisms has been proposed to test metaproteomics pipelines, including seven bacteria and two yeasts [26]. In this case, the genomes of virtually none of the specific microbial strains had been sequenced and publicly released, so the authors supplemented their work with draft genomes and metagenomic sequence data which could be handled with a proteogenomics-derived approach. However, the quantities of these organisms assessed were only approximate in terms of colony-forming units, and because of the large size difference between yeasts and bacteria, the yeast proteomes may have dominated the bacterial proteomes. Another laboratory-assembled microbial mixture (4MUM) was proposed, with an unbalanced ratio but limited to only four bacteria [27]. Based on these pioneering and interesting datasets, the reliability of taxonomic assignment using several tools and various database searches was evaluated [26,27]. Two relatively simplistic hybrid proteomes comprising proteins extracted from *Escherichia coli*, *Saccharomyces cerevisiae*, and human HeLa cells were also proposed for comparison [28,29]. Finally, 3 artificially assembled microbial communities, including 32 archaea, bacteria, eukaryotes, and bacteriophages, were proposed with the quantification of cell numbers by microscopy using a counting chamber [30]. 

Accurate references are crucial for evaluating bioinformatics strategies and tools in the field of proteotyping. Here, we chose to focus our attention on a reference dataset comprising only bacterial proteins and representative of a wide range of phylogenetic distances between members. We assembled a unique consortium and recorded several high-throughput tandem mass spectrometry datasets acquired on individual peptide digests produced from 24 bacteria and their normalized mixture. The dataset can be used to improve bioinformatic tools dedicated to proteotyping microorganisms from complex samples, or to extracting taxonomic or functional information from metaproteomic experiments.

## 2. Results

### 2.1. Assembly of 24 Bacterial Peptide Digests According to a Predefined MS/MS-Responsive Equimolar Ratio

**Figure 1:**
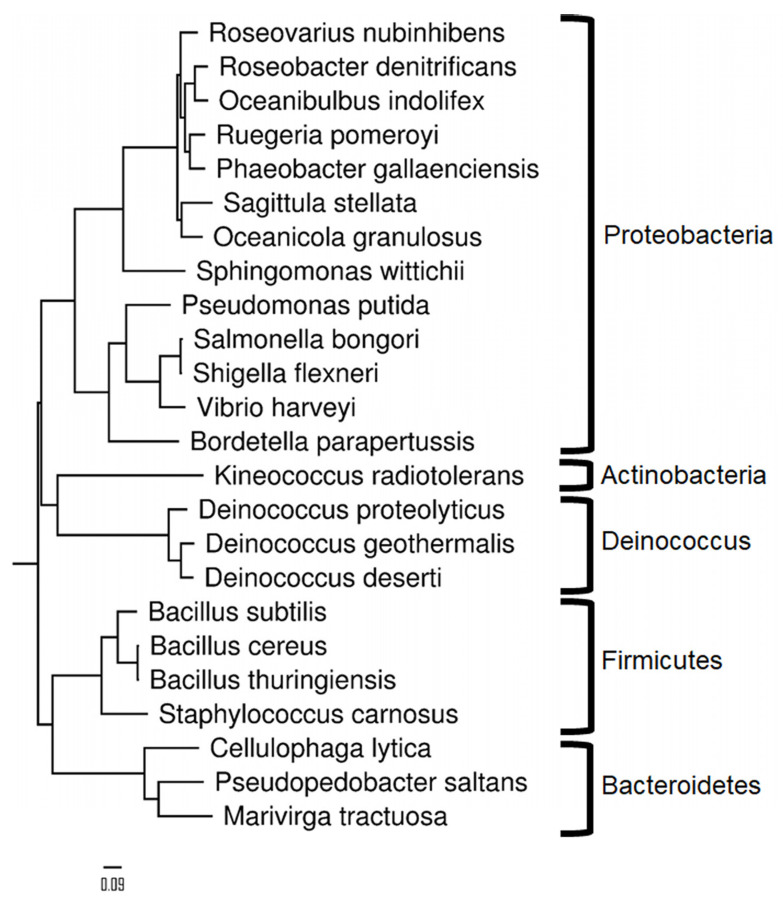
Phylogenetic tree of the 24 species included in the Mix24 assemblage. A multiple alignment of supervectors of COGs from each organism known to be systematically conserved among all organisms was performed using BLAST, clustalW, and GBlocks. The aligned fasta was submitted to PhyML http://phylogeny.lirmm.fr/phylo_cgi/one_task.cgi?task_type=phyml (accessed on 9 May 2023) with default parameters for maximum likelihood distance calculations. FigTree v1.4.3 was used to display the final tree.

Table 1 reports the names and characteristics of the 24 bacterial strains chosen for the microbiota reference resource as representing a large diversity of phylogenetic distances between members, some being closely related and others very distant. These bacteria comprise 24 distinct species representatives of different environmentally or medically relevant microbiomes (marine bacteria, soil bacteria, and human-associated bacteria). This microbiota reference resource includes four clinically important pathogens: *Shigella flexneri*, *Salmonella bongori*, *Bordetella parapertussis*, and *Bacillus cereus*, and bacteria of biotechnological interest (*Staphylococcus carnosus*, *Pseudomonas putida*, and *Sphingomonas wittichii*). Figure 1 shows a phylogenetic tree showing the distances between the different bacterial species. In order to be able to assess whether closely related species can be discriminated from each other, some bacteria belonging to the same genus are included: three Deinococcus and three Bacillus representatives. Two of the Bacillus species, namely, *Bacillus cereus* and *Bacillus thuringiensis*, are very closely related and belong to the so-called “B. cereus group” while presenting different phenotypes and pathogenic effects [31,32]. *Shigella flexneri*, which is known to be difficult to distinguish from *Escherichia coli*, is also included. The proposed reference dataset thus covers 20 genera, 14 families, 13 orders, 9 classes, and 5 phyla (Actinobacteria, Bacteroidetes, Deinococcus-Thermus, Firmicutes, and Proteobacteria). Their genomic repertoires range from 2355 (*Staphylococcus carnosus*) to 6073 (*Bacillus thuringiensis*) protein-encoding genes each. The total number of theoretical polypeptide sequences when merging the 24 organisms is 97,919 sequences, totaling 30,938,543 amino acids. 

As insights into such samples obviously rely on precise quantitative measurements, the mixture was constructed from individual bacterial peptide digests in an exact MS/MS-responsive equimolar ratio. For this, we chose to generate experimental tryptic peptide digests from each bacterium grown in its most favorable condition and normalized by weight to quantify the MS/MS-detectable peptides in standard conditions and to adjust the mixture based on these quantities. Equalizing the amounts of peptides and their mass spectrometry signals for each microorganism prevents any possible bias due to differences in cell disruption and protein extraction yields between bacteria and bias regarding differences in ionizability that could be observed for the peptides from the most-abundant proteins of each bacterium. Furthermore, this procedure allows for the production of normalized batches of any complex peptide mixture when used on a large scale as an inter-laboratory standard. The 24 peptide digests were analyzed by tandem mass spectrometry with a 90 min gradient to assess the numbers of MS/MS-detectable ion spectra, assignable spectra, unique peptides, and validated proteins, as detected with a standard procedure search against each specific genome database. When considering the 24 individual nanoLC-MS/MS runs, a total of 73,366 unique peptide sequences (when I and L residues are equated) were proven to be MS/MS detectable by the LTQ-Orbitrap XL instrument (Supplementary Tables S1 and S2).

### 2.2. Mix24X Datasets 

Tandem mass spectrometry datasets were recorded in data-dependent analysis mode for the Mix24X mixture using two tandem high-resolution mass spectrometers: an LTQ-Orbitrap XL (Thermo) and a Q-Exactive HF (Thermo), both instruments coupled to the same nanoLC chromatographic system. Three analytical replicates were recorded along a 3 h gradient for the first instrument and a 1 h gradient for the second instrument after injecting 315 ng of material. Merging the analytical replicates may give the equivalent of a longer tandem mass spectrometry runtime if needed. Table 2 reports the numbers of acquired MS/MS spectra for these six nanoLC-MS/MS runs. On average, twenty thousand MS/MS spectra were recorded with the first instrument and twice this amount with the second instrument. These datasets were interpreted against a generalist database (NCBInr), resulting in 12% and 21% peptide-to-spectrum matches, respectively, as shown in Table 2. This low assignation rate, compared to those obtained for single species microbial proteomics [33,34], can be explained by two factors. First, the database size is unusually large with 76 million polypeptide sequences. The high peptide sequence diversity of the sample is also rather unusual, as more than 60,000 proteins are present in the sample with a dynamic range typical of bacteria. Such high diversity should inherently increase m/z signal cross-contamination and thus decrease MS/MS spectrum average quality. The higher acquisition speed and discriminative power of the Q-Exactive HF compared to the LTQ-Orbitrap XL instrument results here in an almost two-fold increase in the percentage of MS/MS spectrum assignations. The narrower isolation window for the parent ion in the former instrument (1.6 *m*/*z*) compared to the latter (3.0 *m*/*z*) reduces noisy, simultaneous analysis of co-eluted peptides. The difference in terms of peptide sequences is even more pronounced, with an almost six-fold increase when comparing Q-Exactive HF and LTQ-Orbitrap XL runs. When the runs are merged, a rather quick saturation is observed in terms of peptide sequence discovery for both instruments. Finally, the number of peptide sequences detected when merging the three Q-Exactive HF runs is 9106, while at best, only 1242 could be observed with the LTQ-Orbitrap XL when considering an equivalent acquisition time, i.e., 180 min or 3 × 60 min. 

### 2.3. Taxonomical Characterization Using Species-Specific Peptides

Table 3 shows the numbers and nature of identified genera and species based on unique peptide sequences for two Mix24X datasets acquired with the Q-Exactive HF instrument: a 60 min run and the merge of three 60 min runs. The datasets were queried against the NCBInr database without a priori, and the two lists of peptides were analyzed by the last common ancestor approach. For the 60 min run (Mix24X_HF1), 23 out of the 24 expected bacterial species were identified. It is worth noting that the numbers of species-specific peptides vary over a wide range, as some, such as *Sagittula stellata* and *Sphingomonas wittichii*, are identified through more than 100 species-specific peptides and others via less than 10 peptides. The origin of this discrepancy is linked to the sequencing density of each species. 

**Figure 2:**
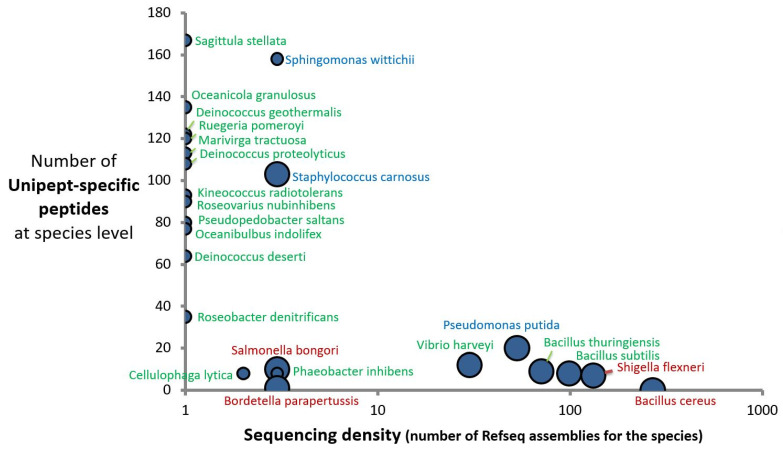
Number of Unipept-specific peptides at the species level as a function of the sequencing density of species and genera. Bacteria are indicated in three colors based on their pathogenicity (red), biotechnological (blue), or environmental (green) relevance. Circle sizes depend on the number of genomes per genera.

**Figure 3:**
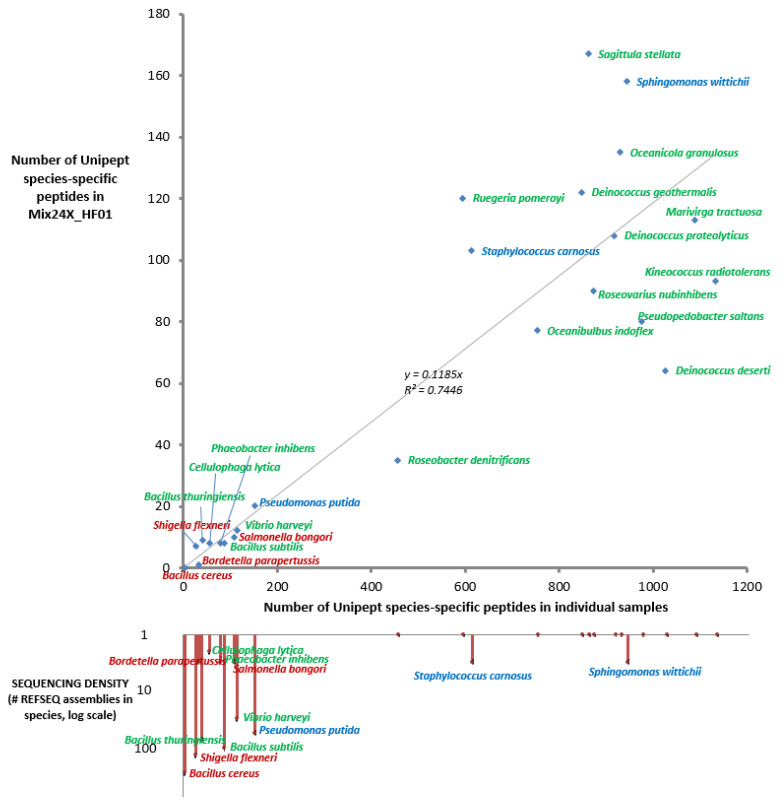
Correlation between Unipept species-specific peptides found in the Mix24 complex mixture and individual proteomes. Bacteria are indicated in three colors based on their pathogenicity (red), biotechnological (blue), or environmental (green) relevance. The simple linear regression parameters are indicated in black. The sequencing density of each species is indicated on the bottom graph.

Figure 2 shows the number of experimental species-specific peptides established for this dataset and the number of strains sequenced for a given species. The sequencing density within each genus is represented proportional to the circle size. An inverse correlation between the two variables is evidenced; the six best-represented species in the database in terms of genome sequences, namely, *B. cereus*, *S. flexneri*, *B. subtilis*, *B. thuringiensis*, *P. putida*, and *Vibrio harveyi*, all have a low number of species-specific peptides. This is also the case at the genus rank, except for the *Staphylococcus carnosus* species, for which numerous distantly related *Staphylococcus aureus* representatives have been sequenced without drastically diminishing the species-unique peptide sequences. As we chose three representatives for each of two genera (Bacillus and Deinococcus), the number of experimental species-specific peptides for these 6 representatives should be lower than for the 18 other bacteria. As shown in Table 3, this is the case for the former (0, 8, and 9 species-specific peptides) but not the latter (64, 108, and 122 species-specific peptides). This difference is due to (i) the higher sequencing density in the genus Bacillus compared to the genus Deinococcus: 2601 versus 31 assemblies, respectively, (ii) the higher number of different genome-sequenced species within the Bacillus genus (203) compared to the Deinococcus genus (23), and (iii) the shorter phylogenetic distances between Bacillus species (*B. cereus* and *B. thuringiensis* distance of 0.0028) compared to the Deinococcus species (*D. proteolyticus* and *D. deserti* distance of 0.086). As a consequence, the sizes of the unique theoretical peptidomes are quite different: 2692 for *B. cereus* ATCC14579, 5404 for *B. thuringiensis* ATCC10792, and 924 for *B. subtilis*, versus 39,261 for *D. deserti* VCD115, 32,003 for *D. proteolyticus* DSM20540, and 31,460 for *D. geothermalis* DSM11300. Thus, the correct identification of a given organism at the species taxonomic rank relies on the number of experimentally detected peptides, the density of genome sequences for a given taxonomic unit, and on taxonomic discriminants defining the species. Figure 3 shows the correlation between the Unipept species-specific peptide sequences observed for the Mix24X_HF01 dataset and those found when LTQ-Orbitrap XL runs have been performed for each individual species and interpreted against the same generalist database, NCBInr. While many more peptides were detected in individual runs (about six-fold more), the percentages of peptides that could be considered as taxon-specific in the mixture or in individual runs are roughly equivalent, whatever the organism.

Due to the dataset size, a threshold of at least two different taxon-specific MS/MS peptides to validate any identification may be defined for removing most of the 25 detected false positives. In such a case, two false negatives have to be considered: *Bacillus cereus* and *Bordetella parapertussis*. With more data to hand, i.e., the merge of three runs acquired with the Q-Exactive HF instrument corresponding to the equivalent of a 3 h acquisition time with the same mass spectrometry platform, a higher number of taxon-specific MS/MS peptide sequences (2310) is obtained (Supplementary Tables S3 and S4). In this case, some false positives with a maximum of two species-specific peptides are evidenced, namely *Vibrio alginolyticus* and *Trypanosoma cruzi*. A threshold of at least three different taxon-specific MS/MS peptides may be proposed to get rid of false-positive identifications for this dataset comprising almost 120,000 MS/MS spectra. In this case, *Bacillus cereus* is identified on the basis of one species-specific peptide and will result in a false negative. As expected, the threshold for validating the identification of species should be adapted to the dataset size.

### 2.4. Identification of Genus and then Species with a Cascade Search

We proposed another strategy consisting of a cascade search: the first search is done to identify the genera present in the sample, and the second search is conducted on a reduced database containing only representatives of the identified genera. As shown in Table 4, the number of genus-specific peptides established by the Unipept tool from the list of MS/MS-detected peptides is rather large (≥10) for the 20 genera present in the Mix24X sample, while false positives only appear when considering a threshold of less than three genus-specific peptides. This is true whatever the dataset under consideration (Mix24X_HF1 or the merge of the three Q-Exactive HF runs). The lowest numbers of genus-specific peptides are observed for Shigella, with 10 and 13, respectively. These low values are logically explained because this genus is closely related to Escherichia and does not have per se numerous taxon-specific peptides. Thus, with the objective of improving the identification of species present in the sample, we considered carrying out a second-round MS/MS search using a database reduced to all the representatives of genera validated with at least three genus-specific peptide sequences in the first round. Applied to the 60 min Q-Exactive HF run (Mix24X_HF01), this procedure led to the identification of 9571 peptide sequences, of which 2272 are considered as species-specific by the Unipept web tool. This list of MS/MS-certified peptides indicated the presence of 25 species when considering a threshold of at least 2 different peptides. In addition to the correct identification of the 24 expected species, *Staphylococcus schleiferi* was also listed. As this species belongs to one of the 20 genera previously identified, this false positive cannot be identified per se. 

## 3. Discussion

Tandem mass spectrometry proteotyping has proven a valuable methodology for the identification of microbial isolates [2,3]. Based on several thousand peptides recorded in a few minutes, identification to the species level is possible as soon as several representatives of that species have been genome sequenced, appropriately annotated, and the results deposited in the database used for interpretation. For a new environmental isolate corresponding to a species of which no member has yet been genome sequenced, the result will indicate the branch of life it belongs to at a higher taxonomical rank and deliver the name of the genome-sequenced species that is phylogenetically closest. With the increase in the coverage of the entire tree of life in terms of genome sequences, the methodology has a promising future. The methodology also has the potential to be highly discriminating and, similar to whole genome sequencing, to highlight differences between strains. In addition, the proteotyping methodology has been shown to be rapid in yielding a result and high throughput, the preparation of samples being easily carried out in 96-well plates [10]. We propose here a dataset acquired on a mixture of 24 microorganisms in order to promote the development of the methodology for more complex samples.

Proteotyping complex samples is a challenge for current proteomics computational tools, as these tools are oriented towards a simple theoretical analysis of the proteome of a single organism in most cases, thus taking into account a database limited to only a few thousand protein sequences. Computational metaproteomics methods are currently being developed with the objective of functional characterization of microbiomes, including taxonomical identification of organisms present in complex samples. The main difficulty with these samples is that they contain many organisms, their exact composition is unknown, and in many cases, the organisms present have not been genome-sequenced and are not even taxonomically characterized to the species or genus level. Importantly, strain-resolved metaproteomics has been proposed for samples containing few strains and for which genome information is available [35]. Here, a strain-resolved metaproteomics strategy will maximize the results from the Mix24 dataset, as all 24 corresponding genomes are available. This should be taken into consideration when comparing results from this standard dataset with those calculated for unknown samples. As noted earlier, the opportunities and challenges for metaproteomics in terms of data extraction from raw files acquired by tandem mass spectrometry are numerous [36,37]. The power of de novo interpretation has also been highlighted to identify variants not yet genome sequenced [38,39]. Although many interesting tools have recently been proposed to address specific metaproteomics questions, there is a clear need to evaluate these computational tools with ground truth standards. Different concepts can also be proposed to speed up bioinformatics processes, such as using custom databases with less information based on non-redundant protein groups or non-redundant taxonomic units for example, or to get a more complete view with larger databases derived from metagenomics or metatranscriptomics experimental data. Here, we describe a metaproteomics reference standard comprising 24 bacterial species and propose several reference datasets that could be very useful for the comparative evaluation of new computational tools. 

Quantitative analysis of taxonomic units, proteins, and, more importantly, functions and pathways is the ultimate goal of metaproteomics for an in-depth comparison of conditions and gain insights into key biological questions [23,40]. Here, the dataset proposed could be used to evaluate label-free quantification methods for taxonomic units. The biomass of organisms at a given taxonomical rank can be assessed on the basis of taxon-specific peptides, but the result is obviously distorted by the density of sequenced genomes, which varies considerably along the branches of the tree of life. Therefore, new approaches must be proposed and tested. For the microbiomes, 16S rRNA gene amplicon sequencing is the most widely used approach to assess their composition and compare conditions [41]. However, this approach is being questioned [42]. Current best practices for this methodology rely on the use of commercial artificial samples with known numbers of ribosomal RNA operons to evaluate errors stemming from the amplification stage, including the extraction of genomic DNA, which is far from equivalent depending on bacterial taxonomical units [43]. Additional significant errors regarding the evaluation of cell counts may arise from the variability in the number of copies of the ribosomal RNA operon per cell. This is because many bacteria have multiple copies of the 16S rRNA gene and multiple copies of the chromosome. Furthermore, the number of copies of the chromosome, i.e., polyploidy, can vary with physiological conditions and bacterial taxonomic units [43,44]. With reliable datasets, such as Mix24, and the development of new data mining strategies, tandem mass spectrometry proteotyping could be an attractive alternative for rapid estimation of the taxonomical composition of a complex sample and evaluation of the biomass ratio of the components. 

In conclusion, the standard Mix24X datasets presented here can help to compare the performance of specialized computational methods for proteotyping and to optimize their parameters. As an example, here, we could easily evaluate false-positive identifications of taxonomic units. Furthermore, normalization of the mass spectrometry signal of the 24 peptide extracts should allow reproducible production of large batches of this reference if required. In principle, the Mix24X reference resource can be used as a control quality standard for the validation of analytical platforms and fine-tuning of acquisition parameters. We concluded that the Mix24 dataset is of great interest to evaluate proteotyping pipelines with a specific worst-case scenario, such as closely related organisms or densely genome sequenced genera and species. The Mix24 dataset could be a ground-truth dataset for evaluating the metaproteomics pipeline and adjusting thresholds for obtaining the best sensitivity in terms of species identification without increasing the number of false positives. 

## 4. Materials and Methods

### 4.1. Microbial Cultures and Samples

Table 1 lists the 24 microbial strains, their origins, and their culture conditions. All microbial cultures were grown in liquid culture under aerobic conditions until the stationary phase, in the most appropriate media and temperature conditions, in a BSL2 safety laboratory. Cells were harvested at the stationary phase in order to achieve the least possible experimental variation between bacterial cultures, their exponential growth rates being by nature quite different. Microbial cultures were kept on ice for 2 h to slow growth, limit protease activity, and obtain all cells in a similar physiological condition, i.e., a cold shock, then harvested by centrifugation. Cell densities were evaluated by means of optical density (OD) measured at 600 nm. Aliquots corresponding to 250 µL of cell suspension at OD 600 nm = 1.0 were centrifuged at 6000× *g* for 5 min. The resulting supernatants were removed, and the cell pellets underwent another round of centrifugation for 2 min to remove residual liquid from the tube wall. Wet pellets were flash-frozen and kept at −20 °C until use.

### 4.2. Protein Extraction and Trypsin Proteolysis

For each organism, a specific volume of LDS1X sample buffer (Invitrogen, Villebon sur Yvette, France) consisting of 106 mM Tris/HCl, 141 mM Tris base, 2% lithium dodecyl sulfate, 10% glycerol, 0.51 mM EDTA, 0.22 mM SERVA Blue G250, 0.175 mM phenol red, buffered at pH 8.5, and supplemented with 2.5% beta-mercaptoethanol was added to the frozen pellet (60 mg of pellet, containing 4.5 × 10^6^ bacteria per mg of material). Samples were heated at 99 °C for 5 min in a thermomixer (Eppendorf, Montesson, France), then subjected to sonication in an ultrasonic bath (VWR ultrasonic cleaner, VWR, Rosny-sous-Bois, France) for 5 min to dissolve all the biological aggregates. The 24 samples were transferred to tubes containing 200 mg silica beads and subjected to bead-beating with a Precellys instrument (Bertin technology, Montigny-le-Bretonneux, France) operated at 6500 rpm for 30 cycles of 20 s separated by 30 s pauses. After cell disruption, the tubes were centrifuged at 16,000× *g* for 40 s. The resulting supernatants were transferred into new tubes and heated at 99 °C for 5 min. Four equal amounts (20 µL) of each of the 24 samples were loaded onto NuPAGE 4–12% Bis-Tris gels (Invitrogen) for a short denaturing electrophoresis migration (5 min) at 200 V in MES/SDS 1X running buffer as previously described [45]. The 96 resulting polyacrylamide bands containing the whole soluble proteomes were processed for in-gel trypsin digestion in the presence of 0.01% ProteaseMAX detergent (Promega, Charbonnières-les-Bains, France) as described [46]. The four peptide samples corresponding to the same bacterium were pooled to equalize possible in-gel proteolysis variations. The Mix24X laboratory-assembly was performed by mixing equal XIC-adjusted volumes of the 24 individual peptide pools taking into account MS/MS ion signals from the most intense peptides (top 11 to 109 peptide intensities).

### 4.3. NanoLC-MS/MS Analysis

Peptides were analyzed either with an LTQ-Orbitrap XL hybrid mass spectrometer (Thermofisher, Villebon sur Yvette, France) or a Q-Exactive HF tandem mass spectrometer (Thermo) that is equipped with an ultra-high-field Orbitrap analyzer. Both spectrometers were coupled to an ultimate 3000 nanoLC system (Thermo). For the first instrument, digests (5 µL) were loaded and desalted online on a reverse phase PepMap100 C18 µ-Precolumn (5 µm, 100 Å, 300 µm i.d. ×5 mm, Thermofisher) and resolved on a nano scale PepMap 100 C18 nano LC column (3 µm, 100 Å, 300 µm i.d. × 50 cm, Thermofisher) at a flow rate of 0.3 µL·min^−1^ with a gradient of CH_3_CN, 0.1% formic acid prior to injection into the ion trap mass spectrometer. Peptides were resolved using either a 90 min gradient from 5% to 40% solvent B (0.1% HCOOH/100% CH_3_CN) and solvent A (0.1% HCOOH/100% H_2_O) or a 180 min gradient from 2.5% to 50% solvent C (0.1% HCOOH/20% H_2_O/80% CH_3_CN) and solvent A (0.1% HCOOH/100% H_2_O). A Top 7 strategy was used for the acquisition of MS/MS, and full scan mass spectra were measured from *m*/*z* 300 to 1800. A scan cycle was initiated with a full scan of high mass accuracy in the Orbitrap analyzer (30,000 resolution), which was followed by MS/MS scans in the linear ion trap on the seven most abundant precursor ions (minimum signal required to set at 10,000 and potential charge states of 2^+^ and 3^+^, with a 10 s dynamic exclusion of previously selected ions. For Mix24X assembly analysis with the Q-Exactive HF system (Thermofisher), peptides (5 µL at 63 ng/µL) were also resolved on a nano scale PepMap 100 C18 nano LC column but using a 60 min gradient from 2.5% to 40% solvent C against solvent A at a flow rate of 0.2 µL min^−1^. In this case, a Top 20 strategy was used for MS/MS spectrum acquisition. MS/MS and full scan mass spectra were measured from *m*/*z* 350 to 1500. An isolation window of 1.6 m/z was used in the quadrupole. A scan cycle was initiated with a full scan of high mass accuracy in the Orbitrap HF analyzer (60,000 resolution) and an AGC target set at 3 × 10^6^, which was followed by MS/MS scans at 15,000 resolutions on the twenty most abundant precursor ions (minimum signal required to set at 15,000 and potential charge states of 2^+^ and 3^+^), with a dynamic exclusion of 10 s. MS/MS was acquired with an AGC target set at 1 × 10^5^.

### 4.4. MS/MS Spectrum Assignment and Protein Identification

Peak lists were automatically generated with the extract_msn.exe data import filter (Thermo), with the following options: minimum mass (400), maximum mass (5000), grouping tolerance (0), intermediate scans (0), and threshold (1000). MS/MS spectra were queried against the NCBInr database [47] with the Mascot Daemon software version 2.5.1 (Matrix Science), with the following parameters: full-trypsin specificity, up to 1 missed cleavage allowed, static modifications of carbamidomethylated cysteine (+57.0215), variable oxidation of methionine (+15.9949), mass tolerance of 5 ppm on parent ions, and mass tolerance on MS/MS of 0.5 Da or 0.02 Da for the LTQ-Orbitrap XL and the Q-Exactive HF instruments, respectively. All peptide matches with a Mascot peptide score below a *p*-value of 0.05 were retained. A protein was considered valid when at least two different peptides were detected. The false-positive rate for protein identification was estimated by a search with a reverse decoy database to be below 0.1% using the same parameters. 

### 4.5. Evaluation of Global Ion Intensity for Each of the 24 Peptide Digests for Mix24X Assembly

The nanoLC-MS/MS data for each individual peptide digest were assigned against each specific theoretical proteome database using MaxQuant software (version 1.5.3.30). The global peptide abundance was assessed based on extracted ion chromatogram (XIC) signals extracted for the identified proteins, using ordered peptide XIC intensities from the MaxQuant peptide output files (combined\txt\peptides.txt, intensity column) and taking into account only non-contaminant (CON_) and non-reverse (REV_) peptides. A total of 100 peptide intensities were summed, excluding the top nine peptides to avoid extreme values.

### 4.6. Taxonomical and Functional Data Analysis

Mix24X interpreted files were exported by Mascot 2.5.1 (Matrix Science, London, UK) with a 0.05 identity *p*-value, 0.05 ion score cut-off, MudPIT option enabled for protein scoring, bold red request, and subset protein request. Proteins were first ordered by MudPIT score, then reordered to gather proteins in groups sharing at least one peptide with I/L equated. Proteins reordered on this basis were then validated only if at least two different peptides were associated with at least one “bold red” peptide. The web-interfaced Unipept tool (http://unipept.ugent.be/, accessed on 9 May 2023) was used to calculate the lowest common ancestor (LCA) of the identified peptides with the following options: equate I and L, filter duplicate peptides, advanced missed cleavage handling [48]. The Unipept unique peptidomes were obtained by means of the Unipept Peptidome Analysis module (http://unipept.ugent.be/peptidome, accessed on 9 May 2023). 

### 4.7. Data Repository

The mass spectrometry proteomic data from the Mix24X standard reference were deposited at the ProteomeXchange Consortium (http://proteomecentral.proteomexchange.org, accessed on 9 May 2023) via the PRIDE partner repository [49] with the dataset identifiers PXD005776 (Q-Exactive HF data), PXD005759, and DOI 10.6019/PXD005759 (LTQ-Orbitrap XL data). The mass spectrometry proteomic data from the 24 individual bacterial strains were deposited with the dataset identifier PXD005728 and DOI 10.619/PXD005728.

## Figures and Tables

**Table 1:** Bacterial strains used in this study and growth conditions.

Strain	Gram Staining ^a^	Source ^b^	Growth Condition ^c^
*Bacillus cereus ATCC 14579*	+	UMR408	LB, 24 h, 30 °C
*Bacillus subtilis ATCC 6633*	+	ATCC	BHI, 24 h, 30 °C
*Bacillus thuringiensis DSM 5815*	+	DSMZ	LB, 24 h, 30 °C
*Bordetella parapertussis Bpp5*	−	Pasteur institute	BHI, 48 h, 30 °C
*Cellulophaga lytica DSM 7489*	−	DSMZ	MB, 24 h, 30 °C
*Deinococcus deserti VCD115*	~	BIAM1	diluted TSB, 24 h, 30 °C
*Deinococcus geothermalis DSM 11300*	~	BIAM1	LB, 48 h, 37 °C
*Deinococcus proteolyticus DSM 20540*	~	BIAM1	LB, 24 h, 30 °C
*Kineococcus radiotolerans SRS30216*	+	DSMZ	PTYG, 72 h, 30 °C
*Marivirga tractuosa DSM 4126*	−	DSMZ	MB, 48 h, 30 °C
*Oceanibulbus indolifex HEL-45*	−	DSMZ	MB, 48 h, 30 °C
*Oceanicola granulosus HTCC2516*	−	DSMZ	MB, 48 h, 30 °C
*Phaeobacter inhibens DSM 17395*	−	DSMZ	MB, 48 h, 30 °C
*Pseudomonas putida mt-2 KT2440*	−	DSMZ	LB, 24 h, 30 °C
*Pseudopedobacter saltans DSM 12145*	−	DSMZ	TSB and extracts, 24 h, 26 °C
*Roseobacter denitrificans OCh 114*	−	DSMZ	MB, 48 h, 30 °C
*Roseovarius nubinhibens ISM*	−	DSMZ	MB, 24 h, 30 °C
*Ruegeria pomeroyi DSS-3*	−	DSMZ	MB, 48 h, 30 °C
*Sagittula stellata E 37*	−	DSMZ	MB, 48 h, 30 °C
*Salmonella bongori NCTC 12419*	−	Pasteur institute	TSB, 24 h, 37 °C
*Shigella flexneri 2a 2457T*	−	Pasteur institute	TSB, 24 h, 30 °C
*Sphingomonas wittichii RW1*	−	DSMZ	LB, 120 h, 30 °C
*Staphylococcus carnosus TM300*	+	DSMZ	TSB, 24 h, 37 °C
*Vibrio harveyi ATCC 14126*	−	BIAM2	PB, 24 h, 26 °C

^a^ Gram-positive (+), Gram-negative (−), unusual Gram along the phylum due to the presence of a thick peptidoglycan layer (~); ^b^ kind gift from Catherine Duport (UMR408), Arjan de Groot (BIAM1), Daniel Garcia (BIAM2); ^c^ Luria Bertani broth (LB), Brain Heart Infusion (BHI), Marine Broth (MB), Peptone-Tryptone-Yeast extract-Glucose medium (PTYG), Trypticase soy broth (TSB), 1/10 TSB+ trace elements (diluted TSB). A total of 10 g TSB+ 2 g Yeast extract + 1 g Beef extract based on DSM medium 948 (TSB and extracts), *Photobacterium* Broth, ATCC medium 101 (PB).

**Table 2:** Mix24X datasets and Mascot analysis against NCBInr.

Reference	Gradient Time (min)	MS/MS Platform	MS/MS Spectra	PSMs	Peptide Sequences	Cumulated PSMs ^a^	Cumulated Peptide Sequences ^b^
Mix24X_XL01	180	LTQ Orbitrap XL	20,641	2464	1242	2464	1242
Mix24X_XL02	180	LTQ Orbitrap XL	19,664	2358	1143	4822	1503
Mix24X_XL03	180	LTQ Orbitrap XL	19,085	2145	1075	6967	1642
Mix24X_HF01	60	Q-Exactive HF	40,768	8363	6201	8363	6201
Mix24X_HF02	60	Q-Exactive HF	38,464	8275	6129	16,638	8043
Mix24X_HF03	60	Q-Exactive HF	38,471	8303	6151	24,941	9106

^a^ psms and ^b^ Peptide sequences were cumulated as follows: XL01 + XL02; XL01 + XL02 + XL03; HF01 + HF02; and HF01 + HF02 + HF03.

**Table 3:** Identification of the species rank of Mix24X bacteria and their label-free quantitation.

Species	HF01 Specific Peptides ^a^	HF01 SC ^b^	HF01 + HF02 + HF03 Specific Peptides ^a^	HF01 + HF02 + HF03 SC ^b^
*Bacillus cereus*	0	0	1	1
*Bacillus subtilis*	8	9	10	24
*Bacillus thuringiensis*	9	8	12	27
*Bordetella parapertussis*	1	1	3	5
*Cellulophaga lytica*	8	8	12	21
*Deinococcus deserti*	64	83	99	275
*Deinococcus geothermalis*	122	147	180	428
*Deinococcus proteolyticus*	108	141	153	414
*Kineococcus radiotolerans*	93	90	143	279
*Marivirga tractuosa*	113	126	156	377
*Oceanibulbus indolifex*	77	108	116	312
*Oceanicola granulosus*	135	137	191	379
*Phaeobacter inhibens*	8	12	14	40
*Pseudomonas putida*	20	18	25	54
*Pseudopedobacter saltans*	80	69	128	211
*Roseobacter denitrificans*	35	36	49	101
*Roseovarius nubinhibens*	90	108	126	287
*Ruegeria pomeroyi*	120	148	173	449
*Sagittula stellata*	167	194	242	559
*Salmonella bongori*	10	11	14	37
*Shigella flexneri*	7	7	9	17
*Sphingomonas wittichii*	158	182	208	506
*Staphylococcus carnosus*	103	91	159	278
*Vibrio harveyi*	12	9	23	33
OTHER BACTERIA ^c^	17 (17)	15 (17)	39 (38)	43 (38)
ARCHAEA ^c^	1 (1)	1 (1)	1 (1)	1 (1)
EUKARYOTA ^c,d^	8 (8)	7 (8)	17 (16)	22 (16)

^a^ Species–specific peptides proposed by Unipept; ^b^ Spectral counts assigned to species-specific peptides (Unipept peptide sequences that do not match experimental peptides are not counted); ^c^ Number of different species are indicated into brackets; ^d^ Eukaryota counts do not include mammalian taxonomic units as these are considered as contaminants.

**Table 4:** Identification at the genus rank of Mix24X bacteria and their label-free quantitation.

Genus	HF01 Specific Peptides ^a^	HF01 SC ^b^	H01 + HF02 + HF03 Specific Peptides ^a^	H01 + HF02 + HF03 SC ^b^
*Bacillus*	38	40	50	124
*Bordetella*	83	84	120	247
*Cellulophaga*	121	123	168	333
*Deinococcus*	420	505	624	1546
*Kineococcus*	93	90	143	279
*Marivirga*	113	126	156	377
*Oceanibulbus*	77	108	116	312
*Oceanicola*	135	137	191	379
*Phaeobacter*	73	92	103	262
*Pseudomonas*	52	58	74	175
*Pseudopedobacter*	80	69	128	211
*Roseobacter*	77	73	85	208
*Roseovarius*	94	112	133	292
*Ruegeria*	125	150	179	454
*Sagittula*	167	194	242	559
*Salmonella*	27	30	35	95
*Shigella*	10	11	13	31
*Sphingomonas*	167	191	223	537
*Staphylococcus*	162	153	236	459
*Vibrio*	108	104	173	329
OTHER BACTERIA ^c^	17 (16)	14 (16)	39 (37)	39 (37)
ARCHAEA ^c^	1 (1)	1 (1)	1 (1)	1 (1)
EUKARYOTA ^c,d^	8 (8)	7 (8)	18 (17)	23 (17)

^a^ Genus-specific peptides proposed by Unipept; ^b^ Spectral counts assigned to genus-specific peptides (Unipept peptide sequences that do not match to experimental peptides are not counted); ^c^ Number of different species are indicated into brackets; ^d^ Eukaryota counts do not include mammalian taxonomic units as these are considered as contaminants.

## Data Availability

Data are available in supplementary Tables S1–S4 provided together with the main publication. The mass spectrometry proteomic data from the Mix24X standard reference were deposited at the ProteomeXchange Consortium (http://proteomecentral.proteomexchange.org, accessed on 9 May 2023) via the PRIDE partner repository [47] with the dataset identifiers PXD005776 (Q-Exactive HF data) and PXD005759 and DOI 10.6019/PXD005759 (LTQ-Orbitrap XL data). The mass spectrometry proteomic data from the 24 individual bacterial strains were deposited with the dataset identifier PXD005728 and DOI 10.619/PXD005728.

## Supplementary Materials

The following supporting information can be downloaded at: https://www.mdpi.com/article/10.3390/ijms24108634/s1.
